# Oscillatory Mechanisms of Stimulus Processing and Selection in the Visual and Auditory Systems: State-of-the-Art, Speculations and Suggestions

**DOI:** 10.3389/fnins.2017.00296

**Published:** 2017-05-26

**Authors:** Benedikt Zoefel, Rufin VanRullen

**Affiliations:** ^1^Université Paul SabatierToulouse, France; ^2^Centre de Recherche Cerveau et Cognition (CerCo), Centre National de la Recherche Scientifique, University of Toulouse, UMR5549Toulouse, France; ^3^Nathan Kline Institute for Psychiatric ResearchOrangeburg, NY, United States

**Keywords:** oscillation, attention, perception, alpha, entrainment

## Abstract

All sensory systems need to continuously prioritize and select incoming stimuli in order to avoid overflow or interference, and provide a structure to the brain's input. However, the characteristics of this input differ across sensory systems; therefore, and as a direct consequence, each sensory system might have developed specialized strategies to cope with the continuous stream of incoming information. Neural oscillations are intimately connected with this selection process, as they can be used by the brain to rhythmically amplify or attenuate input and therefore represent an optimal tool for stimulus selection. In this paper, we focus on oscillatory processes for stimulus selection in the visual and auditory systems. We point out both commonalities and differences between the two systems and develop several hypotheses, inspired by recently published findings: (1) The rhythmic component in its input is crucial for the auditory, but not for the visual system. The alignment between oscillatory phase and rhythmic input (phase entrainment) is therefore an integral part of stimulus selection in the auditory system whereas the visual system merely adjusts its phase to upcoming events, without the need for any rhythmic component. (2) When input is unpredictable, the visual system can maintain its oscillatory sampling, whereas the auditory system switches to a different, potentially internally oriented, “mode” of processing that might be characterized by alpha oscillations. (3) Visual alpha can be divided into a faster occipital alpha (10 Hz) and a slower frontal alpha (7 Hz) that critically depends on attention.

## Introduction

Imagine looking for someone in a crowd, trying to keep the person's characteristics in mind while suppressing other, potentially distracting events: Constantly bombarded with a continuous stream of sensory information, our brain needs to select, filter and prioritize: the use of top-down processes for this task is indispensable. Recent research suggests that *neural oscillations*, rhythmic fluctuations in the excitability of neural populations, are the brain's key feature in these processes: Events that coincide with the oscillation's high excitability phase are amplified whereas events occurring during the low excitability phase are suppressed, and the brain seems to use this mechanism as a powerful tool to gate and filter input (Schroeder and Lakatos, 2009). This mechanism can also be seen as a way of environmental subsampling: “Snapshots” of the environment are taken at a rate that corresponds to the frequency of the respective oscillation and the moment of the “snapshot” might be optimized by an alignment of neural oscillations with external events (for a review, see VanRullen et al., 2014). Moreover, the oscillatory power can impact the overall responsiveness of a given brain region, a mechanism that has been associated with a modulation of the neural firing rate (Haegens et al., 2011b; Jensen et al., 2012).

An important role of neural oscillations for attentional selection and stimulus processing^1^ has been shown across modalities: For the visual (Lakatos et al., 2008), auditory (Stefanics et al., 2010), somatosensory (Haegens et al., 2011b), motor (Arnal, 2012), and olfactory systems (Kay, 2014). Although the basic mechanisms, common across modalities, are relatively well understood (Schroeder and Lakatos, 2009; Arnal and Giraud, 2012; Calderone et al., 2014), there seem to be differences in oscillatory mechanisms of stimulus selection between modalities whose systematic investigation began only recently (Thorne and Debener, 2014; VanRullen et al., 2014). In this paper, we will contrast the two modalities that are arguably the most important for human perception and behavior: vision and audition. Recently, it has been suggested that these modalities are confronted with different requirements for stimulus processing, largely due to fundamental differences in the input the two systems receive: Whereas visual input is relatively stable in time and might not require processing that is precise on a millisecond time scale, auditory input changes rapidly and relies crucially on a processing system that can cope with fast-fluctuating information (Thorne and Debener, 2014; VanRullen et al., 2014; Zoefel et al., 2015). Here, we go one step further and summarize and discuss differences in the oscillatory mechanisms underlying stimulus processing and selection in vision and audition. We argue that these differences are a direct consequence of the requirements imposed on each system by the particular input. We start by giving an overview of oscillatory frequencies involved in stimulus processing and selection in the two systems (Section “Frequencies of Stimulus Processing: Summary”). In the core of this article (Section “Relation to the System's Input”), we then structure these findings systematically, based on different properties (timing, predictability, and salience) of the stimulus input, and on consequences of these properties for oscillatory processes. This section is guided by several questions: Can the two systems adapt to their environment—and do they even need to? Do oscillatory mechanisms depend on whether the stimulus is rhythmic (arguably the preferred case for oscillatory processing as an alignment between oscillation and stimulus is possible) or only a single event (Section “Adjustment vs. Entrainment”)? What happens when the input is unpredictable or unattended (Section “Processing “modes””)? Answering these questions has critical implications for our understanding of neural oscillations involved in attention and stimulus selection. As we will see, significant progress has been made in recent years, but new questions arise with the increased knowledge. Those questions are also addressed in this paper. Several hypothetical answers are provided, based partly on previous findings and partly, as we emphasize here, on speculation. Experimental approaches that are necessary to investigate the proposed hypotheses are also discussed.

## Frequencies of stimulus processing: summary

**Figure 1:**
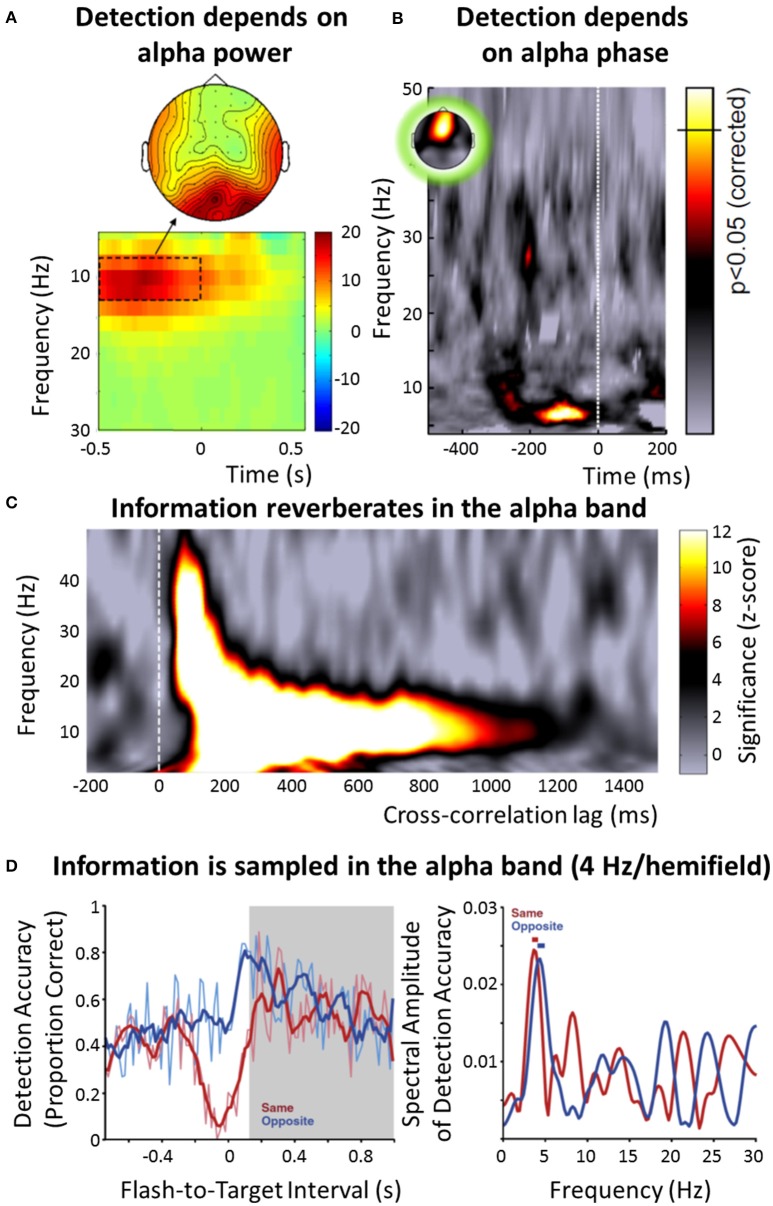
**Overview of the role of neural oscillations for stimulus selection and processing in vision**. **(A)** Difference in EEG power (color-coded) around target onset between subjects that did not perceive near-threshold visual targets and those that did (reproduced with permission from Hanslmayr et al., 2007). Results indicate that visual detection depends on alpha power, with lower power leading to an improved detection. **(B)** Detection of a weak visual target also depends on the phase of the alpha band, as measured in the EEG (reproduced with permission from VanRullen et al., 2014, the original data is presented in Busch et al., 2009). The strength of modulation of target detection by the EEG phase in the respective frequency band is color-coded; the significance threshold is marked on the color bar. **(C)** When a random luminance sequence is presented to human subjects and their EEG is recorded in parallel, a reverberation (“perceptual echo”) of this visual information can be found in the electrophysiological signal for up to 1 s (using cross-correlation between luminance sequence and EEG), but only in the alpha band (reproduced with permission from VanRullen et al., 2014, the original data is presented in VanRullen and Macdonald, 2012). **(D)** After a visual stimulus cues attention to one visual hemifield, the probability of detecting a succeeding target fluctuates rhythmically, and in counterphase depending on whether the target occurred in the same or opposite hemifield (left; reproduced with permission from Landau and Fries, 2012). This “visual rhythm” fluctuates at 4 Hz per visual hemifield (right), indicating an overall sampling rhythm of 8 Hz, thus lying within the alpha band. Note that some effects **(A,C)** seem to have a somewhat higher frequency than others **(B,D)**, leading to the distinction between an “occipital alpha” (~10 Hz) and a “frontal alpha” (~7-8 Hz) in this paper (following VanRullen, 2016).

There is overwhelming evidence for the alpha band (7–13 Hz) as the principal frequency range of stimulus processing in the visual system (Figure 1). This observation was already published by Berger (1929) who reported a dependence of alpha power on the visual input: Alpha power in the electroencephalogram (EEG) increases when subjects close their eyes. Since then, both theoretical and experimental approaches provided convincing evidence that the alpha band is related to an inhibition (or disengagement) of brain regions (Klimesch et al., 2007; Jensen and Mazaheri, 2010; Foxe and Snyder, 2011): For instance, alpha power increases in the hemisphere that is ipsilateral to an attended stimulus (and therefore less strongly involved in its processing) (Thut et al., 2006; Sauseng et al., 2009), or in brain regions not involved in the current task (Zumer et al., 2014). Moreover, it has been shown that visual perception is directly related to the alpha band: The detection of a visual target depends on alpha power (Hanslmayr et al., 2007; Romei et al., 2008; Figure 1A). EEG alpha phase impacts both the probability of detecting a visual target and the likelihood of perceiving a phosphene during transcranial magnetic stimulation (TMS) (Busch et al., 2009; Mathewson et al., 2009; Dugué et al., 2011; Figure 1B), and random visual input seems to reverberate in the brain at a frequency corresponding to the alpha band (VanRullen and Macdonald, 2012; Figure 1C). Similarly, when systematically testing a wide range of physiologically plausible frequencies, the strongest neural resonance in response to rhythmic visual input (e.g., as steady-state response) is observed in the alpha band (Herrmann, 2001; de Graaf et al., 2013), and a longer-lasting manipulation of neural activity by electric current has mostly been reported in that frequency range (e.g., an increased power can be observed several minutes after the stimulation; Thut and Miniussi, 2009; Zaehle et al., 2010). A single pulse of TMS induces a reverberation of endogenous alpha oscillations, but of no other frequency bands (Herring et al., 2015). Together, these findings might indicate that the intrinsic frequency of neurons and/or neuronal circuits (Hutcheon and Yarom, 2000) in the visual system is indeed located predominantly in the alpha band. Finally, both the probability of detecting a visual stimulus after a cue (Figure 1D) and the following reaction time fluctuate periodically (Landau and Fries, 2012; Fiebelkorn et al., 2013; Song et al., 2014). In these studies, the perceptual and behavioral fluctuations have been found at a frequency of 4 Hz *per visual hemifield*, with the two 4 Hz rhythms in opposite phase, indicating an overall rhythmicity of 8 Hz, and thus lying within the alpha band (Zoefel and Sokoliuk, 2014).

Following recent work by VanRullen (2016), one important distinction should be made here: Whereas some studies report effects in the alpha band around 10 Hz, linked to a topographical distribution that is centered on the occipital lobe (e.g., Figures 1A,C), the peak frequency of the effect described in other studies seems to be somewhat lower and located in more frontal^2^ regions (7–8 Hz; e.g., Figures 1B,D). Indeed, a systematic compilation of different studies investigating the role of EEG phase for perception yielded prominent effects at two different frequencies, 7 Hz and 11 Hz (see Figure I in VanRullen, 2016). In a recent study, Keitel and Gross (2016) applied sophisticated signal analysis methods to resting-state magnetoencephalography (MEG) data in order to characterize the spectral profile (termed “spectral fingerprints”) measured in different brain regions. Interestingly, they demonstrated a clear 10 Hz (but no 7 Hz) peak in occipital regions, and a 7 Hz (but no 10 Hz) peak in Inferior Frontal Gyrus. It is thus likely that the two types of effects stem from different generators of oscillatory processing (VanRullen, 2016), a point that we will return to in the following sections. Also, it is unclear whether a frequency of 7–8 Hz can be assumed to reflect “textbook alpha” (or whether it is rather part of the theta band)—nevertheless, for the sake of simplicity, in the following, we will designate both bands as “alpha,” but differentiate between an “occipital alpha” (~10 Hz) and “frontal alpha” (~7–8 Hz).

Although neural activity in the gamma band (~30–70 Hz) has often been reported in the visual system, gamma band power might be tightly linked (“coupled”) to the phase of the alpha band (Bahramisharif et al., 2013; Roux et al., 2013; Jensen et al., 2014): Indeed, the 8-Hz periodicities observed in visual detection performance seem to be correlated with changes in gamma power that fluctuate at the same rhythm (Landau et al., 2015). Gamma activity is often associated with bottom-up processing of sensory information and is present across sensory systems (Fontolan et al., 2014; Bastos et al., 2015). In this paper, we focus on slower frequency bands associated with top-down components of stimulus processes (e.g., attentional selection or predictions) and refer instead to comprehensive reviews published on gamma oscillations in the brain (Fries et al., 2007; Ray and Maunsell, 2015).

**Figure 2:**
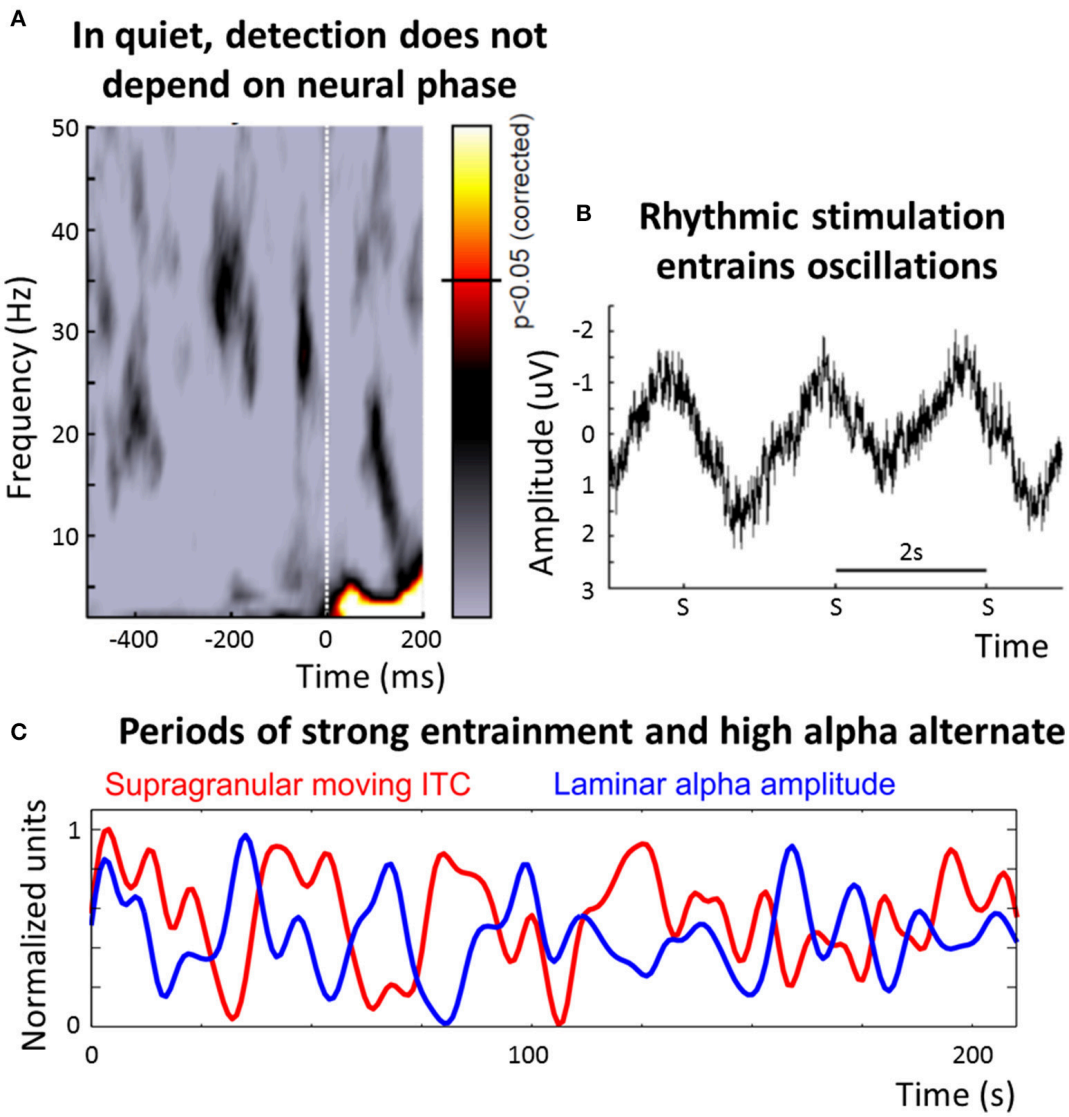
**Overview of the role of neural oscillations for stimulus selection and processing in audition. (A)** Detection of a near-threshold target is independent of the EEG phase when presented in quiet (reproduced with permission from VanRullen et al., 2014; the color-code corresponds to that in Figure 1B). **(B)** It is a widespread phenomenon that oscillations entrain to rhythmic auditory stimulation. Shown is the data from a study in which a train of pure tones, with a repetition rate of 0.5 Hz, has been presented to human subjects, and the EEG was recorded in parallel (reproduced with permission from Zoefel and Heil, 2013). The amplitude of the tones was set to a near-threshold level and subjects had to press a button whenever a tone was detected; the plot shows EEG data, averaged across subjects, in response to three subsequently missed targets (denoted “S”). An oscillatory signal, entrained to the rhythmic stimulation, is apparent—as subjects did not consciously perceive the stimulation, a potential contamination by evoked potentials introduced by the stimulation is minimized. **(C)** The auditory system seems to be able to switch between a “rhythmic mode.” in which processing is determined by oscillations corresponding to the input rate of the entraining stimulus, and an “alpha mode,” in which alpha oscillations dominate the processing. During rhythmic stimulation, large fluctuations in the amount of phase entrainment (indicated by the amount of phase-locking in moving time windows of 5 s, shown in red) and alpha power (blue) exist (reproduced with permission from Lakatos et al., 2016). Importantly, periods of pronounced entrainment and of high alpha power alternate, suggested by a phase opposition between the two functions. This finding was interpreted as alternating periods of external and internal attention. In this paper, we hypothesize that processing in the “alpha mode” might be generalized to input in which no regular structure can be detected, and this speculation requires further experiments (cf. Box 2). ITC, inter-trial coherence.

The dominant frequency of stimulus processing in the auditory system is less clear than in the visual one (Figure 2): On the one hand, many studies describe an alignment between the phase of neural oscillations in the delta/theta band (~1–8 Hz) and rhythmic stimulation (Lakatos et al., 2008; Schroeder and Lakatos, 2009; Stefanics et al., 2010) and this alignment can decrease reaction time (Stefanics et al., 2010), increase efficiency of stimulus processing (Cravo et al., 2013) and seems to be present even after stimulus offset (Lakatos et al., 2013; Hickok et al., 2015). Phase entrainment can also be observed when subjects do not consciously perceive the stimulus, ruling out contamination by evoked potentials (Zoefel and Heil, 2013; Figure 2B).

On the other hand, the alpha band seems to be important as well (Obleser et al., 2012; Strauß et al., 2014; Weisz and Obleser, 2014): Alpha power can be modulated by auditory attention like in the visual system (Kerlin et al., 2010; Frey et al., 2015), speech intelligibility co-varies with alpha power (Obleser and Weisz, 2012; Wöstmann et al., 2015), and the phase of the alpha band modulates auditory stimulus detection if entrained by transcranial alternating current stimulation (tACS; Neuling et al., 2012). Moreover, the power of the gamma band can be coupled to most frequency bands (Lakatos et al., 2005; Fontolan et al., 2014; however we note that, to our knowledge, alpha-gamma coupling has yet to been shown in the auditory system).

Although the auditory system seems to “resonate” (e.g., as steady-state response to a rhythmic stimulus) most strongly in the 40-Hz (i.e., gamma) range (Galambos et al., 1981), several studies suggest that similar phenomena can be found in lower frequency bands as well (e.g., Liégeois-Chauvel et al., 2004). Moreover, human auditory perception is most sensitive to amplitude fluctuations and frequency modulations at a frequency of ~4 Hz. This has been demonstrated in a multitude of psychophysical experiments using a wide range of stimuli (e.g., amplitude- or frequency-modulated tones or noise) and measures (e.g., discrimination thresholds), and has been summarized extensively by Edwards and Chang (2013). Thus, it is difficult to determine a distinct frequency of stimulus processing in the auditory system (indeed, there are “spectral fingerprints” at many different frequencies in Superior Temporal Gyrus, the location of auditory cortices; Keitel and Gross, 2016). Instead, the auditory system might utilize different frequencies for different purposes, and the reported results have to be interpreted in tandem with the respective stimulation protocol, as argued in the following section.

## Relation to the system's input

### Adjustment vs. entrainment

In the following sections, a critical point is the differentiation between adjustment and entrainment. Whereas we define *adjustment* to a stimulus as an adaption of oscillatory parameters to the timing of an anticipated (often single) event, *entrainment* involves an (additional) inherent regularity of the stimulus to which the oscillation can be aligned. For example, it would be possible to adjust (but not entrain) the oscillatory phase to the moment a well-known traffic light expectedly turns green, and the regular siren of a passing ambulance could entrain the phase of oscillations.

The alpha band seems to be the dominant frequency of stimulus processing in the visual system both in the presence (Herrmann, 2001; de Graaf et al., 2013) and absence (Berger, 1929; Busch et al., 2009; VanRullen and Macdonald, 2012) of rhythmicity in the environment. Alpha oscillations in the visual system have been found to adjust when the onset or spatial location of expected upcoming events is known, but no external rhythm is present: For instance, the alpha lateralization effect described above is influenced by the predictability of the spatial location of the target, indicating an active adjustment of alpha power based on anticipatory spatial attention (Gould et al., 2011; Haegens et al., 2011a; Horschig et al., 2014). Alpha power is also adjusted when both timing and spatial location of the visual target is known (Rohenkohl and Nobre, 2011). The described attentional modulation of alpha power is correlated with the predictability of an upcoming visual stimulus (Bauer et al., 2014), indicating an involvement of alpha oscillations in predictive processes. Finally, Bonnefond and Jensen (2012) showed an adjustment of both alpha power and phase prior to the expected onset of a distractor in a visual working memory task, and Samaha and colleagues (Samaha et al., 2015) demonstrated an improvement in performance in a visual discrimination task when the alpha phase was adjusted to the expected target onset (but see van Diepen et al., 2015, for a negative finding).

In the absence of regular stimulus timing (indeed, stimulus timing was predictable, but not rhythmic in Gould et al., 2011; Haegens et al., 2011a; Rohenkohl and Nobre, 2011; Bonnefond and Jensen, 2012; Bauer et al., 2014; Horschig et al., 2014; Samaha et al., 2015), there is not much evidence of other frequency bands adjusting to expected events or location, indicating that the alpha band is indeed the preferred frequency of stimulus processing for the visual system. It is of note that, of course, rhythmic stimuli (such as visual flicker) at non-alpha frequencies introduce a rhythmic component in the recorded signal whose frequency corresponds to the stimulation frequency (i.e., steady-state evoked potentials; Herrmann, 2001) and phase entrainment has been demonstrated for the visual system (Lakatos et al., 2008; Spaak et al., 2014; Gray et al., 2015). However, evidence for phase entrainment at frequencies beyond the alpha band remains sparse—for instance, steady-state potentials obtained in response to flicker show a prominent peak at 10 Hz (Herrmann, 2001)—and is often paired with auditory stimulation. Moreover, in contrast to the auditory system, visual events are rarely cyclic (indeed, flickering stimuli are rare in a natural visual environment), but rather restricted to a specific moment in time^3^. Based on this notion, we suggest that, instead of entraining, the visual system mostly *adjusts* its oscillations to upcoming events. Interestingly, in line with our suggestion, a recent study by Breska and Deouell (2017) showed that a rhythmic visual stream does not lead to a higher EEG phase concentration at an expected stimulus onset (based on the stimulus rhythm) than a non-rhythmic visual stream that also leads to predictions about upcoming events, indicating that temporal predictions in the visual system might not benefit from an additional rhythmic component in the stimulus input. Nevertheless, we acknowledge that this notion remains speculative until more experimental data has been collected; it is therefore discussed in more detail in Box 1 and in the final section of this article. A phase-reset prior to or at the moment of the expected event might be an important tool for this adjustment (Canavier, 2015). Another possibility is that the visual system does not prioritize adaptation to stimulation in time, but rather in the spatial domain. It might therefore be more important for the visual system to precisely localize its oscillations (for instance by changing the speed of a traveling alpha wave; Bahramisharif et al., 2013) rather than to change their frequency, as the latter is, by definition, a temporal parameter. Thus, whereas phase entrainment might be an important and highly developed tool for the auditory system (as outlined below), this might not be the case for the visual one.

Box 1 **Speculations, open questions and how to test them.**

**Adjustment vs. Entrainment**

- It is critical to find a way to differentiate “true” entrainment (i.e., an oscillatory mechanism that includes predictions about the *rhythm* of the upcoming stimulation) from “adjustment” (also including predictions, but rather about a single event without inherent rhythm) and a mere regular repetition of evoked neural activity by the rhythmic stimulation. One way to disentangle entrainment from the other two variations would be a demonstration of the alignment of neural oscillations to, or a modulation of behavior by, the expected rhythm after stimulus offset. Indeed, some studies already provided promising results (Gray et al., 2015; Hickok et al., 2015). However, it also needs to be shown that oscillatory signals or behavior measured after stimulus onset are not simply a reverberation introduced by a phase-reset of brain oscillations by the last stimulus: Indeed, in particular in the visual domain, periodic fluctuations of performance can already be observed in response to a single cue (Landau and Fries, 2012; Song et al., 2014) or after non-rhythmic stimulation (Spaak et al., 2014).
- Further studies are necessary that systematically test the impact on neural oscillations in the two systems when rhythmic stimuli (evoking entrainment) or non-rhythmic, but predictable stimuli (evoking adjustment) are presented, potentially combining electrophysiological and behavioral measurements. It would also be interesting to see the outcome when visual and auditory stimuli are combined (see next point).
- Although beyond the scope of this paper, auditory stimuli affect activity in the visual system, and vice versa (Lakatos et al., 2009; Thorne et al., 2011; Ten Oever et al., 2014; van Wassenhove and Grzeczkowski, 2015). Indeed, visual stimulation improves phase entrainment to speech sound (Zion Golumbic et al., 2013)—interestingly, it has not yet been shown that speech sounds can entrain visual cortices in turn. The oscillatory mechanisms involved in these cross-modal processes represent another exciting field of research—for instance, it needs to be determined whether stimuli of another modality can merely phase-reset (i.e., *adjust*) oscillations in primary cortical regions of a given modality, or whether “true” phase entrainment is involved. A recent suggestion emphasized the directionality between modalities, with preceding sound alerting the visual stimulation about subsequent input, and preceding visual stimulation preparing the auditory system about the exact timing of upcoming events (Thorne and Debener, 2014).

***“Occipital Alpha” vs. “Frontal Alpha” in The Visual System***.

- As described throughout this article, there is relatively clear evidence of a distinction between a faster occipital, and a slower frontal alpha. However, both the functional roles and the origins of these two types of alpha oscillations are poorly understood. It needs to be determined (1) whether these rhythms can co-exist, (2) how and where they are generated, and (3) whether the term “frontal alpha” is justified or whether “frontal theta” would be more appropriate (and if yes, why). Experimental paradigms are needed in which subjects' attentional resources can be modulated in a controlled way: According to our hypothesis, occipital alpha would play a most pronounced role in regions or tasks in which external attention is weak, and frontal alpha would affect behavior most strongly in tasks in which visual attention is focused.
- When a random luminance is presented, the presented visual information seems to reverberate in the EEG at a frequency of ~10 Hz, reflecting occipital alpha (VanRullen and Macdonald, 2012). Interestingly, attention does not change this frequency to 7 Hz, as it might be expected from the hypothesis described here, but rather enhances the observed “echo” at 10 Hz (VanRullen and Macdonald, 2012). This non-trivial finding might indicate that occipital alpha can persist during an attentional state in certain cases: how the different factors (occipital alpha, frontal alpha, and attention) interact is an exciting topic for future research.

In contrast to the visual system, time is one of the most important features for the auditory system (Kubovy, 1988; VanRullen et al., 2014). The need for the auditory system to adapt to the temporal structure of its input might thus be greater than for the visual one. As shown in psychophysical experiments (VanRullen et al., 2014), “blind” subsampling of the environment might not be possible for the auditory system, as the temporal structure of the input might be destroyed. Due to this increased demand of temporal flexibility, the auditory system might make use of the different temporal scales provided by the brain: Neural oscillations cover a wide temporal range (Buzsáki and Draguhn, 2004; Lopes da Silva, 2013), cycling at intervals between seconds (infraslow, 0.1 Hz) and several milliseconds (high gamma range, >60 Hz). Moreover, auditory stimuli are often rhythmic, making neural oscillations a valuable and convenient tool for synchronization with the environment (Schroeder and Lakatos, 2009). This notion might explain the variety of findings described in the previous section: In contrast to the visual system, the frequency of operation might strongly depend on the input to the system in the auditory case.

Many environmental sounds, including speech sounds, contain amplitude fluctuations in the range of the delta/theta band. It is possible that one of the “preferred” rhythms of the auditory system includes this frequency range (Edwards and Chang, 2013), explaining the multitude of studies reporting an alignment of delta/theta oscillations with environmental rhythms. In a multi-speaker scenario or when speech is mixed with noise, the alignment between these oscillations and the envelope of speech is increased for attended speech, suggesting a mechanism of auditory stream selection (Ding and Simon, 2013; Zion Golumbic et al., 2013). Entrainment to speech persists even when slow spectral energy fluctuations have been removed, and this phenomenon can be observed in both humans and non-human primates (Zoefel and VanRullen, 2015a,b,c; Zoefel et al., 2017). Thus, as suggested before (e.g., Schroeder and Lakatos, 2009), phase entrainment might be one of the key features of stimulus selection in the auditory system.

If no regular temporal structure is present but the onset of an expected auditory target is known, some studies have reported an adjustment of alpha power to the target (reviewed in Strauß et al., 2014): For instance, temporal cues in an auditory working memory task can decrease alpha power (Wilsch et al., 2014) and the expectation of a lateralized auditory target increases ipsilateral alpha power (Müller and Weisz, 2012), similar as described above for the visual system. Nevertheless, evidence remains sparse and most paradigms have focused on multimodal or (audio)spatial attention (reviewed in Foxe and Snyder, 2011). A single study (Ten Oever et al., 2015) reported an adjustment of the phase of low-frequency oscillations to the expected onset of an auditory target, but it is unclear whether the effect is specific to their experimental paradigm, as the cycle length of the concerned oscillations corresponded directly to the time window of target occurrence; indeed, a recent study (van Diepen et al., 2015) did not observe an adjustment of phase to expected auditory stimuli. Thus, further experimental evidence is needed to decide whether the auditory system adjusts its oscillations to expected input even if the latter is non-rhythmic—and, if yes, at what frequency this adjustment takes place.

### Processing “modes”

It has recently been shown that perception in the visual system is relatively robust against a discrete sampling of its input: “Blindly” subsampling (i.e., taking “snapshots” independently of the input's content) videos of sign language on a level that corresponds to the very input of the visual system (i.e. on a frame level) is not particularly harmful to visual recognition performance, even at low subsampling frequencies (<10 Hz), and much less disruptive for performance than a corresponding subsampling procedure for the auditory system (VanRullen et al., 2014). Thus, the visual system might maintain its rhythm of stimulus processing even when it cannot be adjusted, such as during an unpredictable sequence of events.

If phase entrainment is impossible, due to non-rhythmic and unpredictable stimulation (or due to an absence of attention, see below), the “use” of low-frequency oscillations might be detrimental for auditory processing as the timing of upcoming input is unknown (this would result in the “blind” subsampling of the environment mentioned above; VanRullen et al., 2014). In this case, the auditory system might need to change its “mode” of processing, from a mode that is tuned to the temporal structure of the input (with a bias for lower frequencies, due to their dominance in the auditory environment) to another mode, potentially internally oriented to avoid loss of information by oscillatory subsampling on early sensory levels. Interestingly, these two modes resemble two cortical states of primary auditory cortex that have recently been described (Pachitariu et al., 2015): A “synchronized state” that is relatively independent of sensory input and a “desynchronized state,” where the processing of input sounds is precise and reliable (corresponding to an “entrainment” or “rhythmic mode”). Recently, important experimental evidence for an internally oriented auditory mode of processing was reported by Lakatos et al. (2016). The reported data suggest that, in this mode, alpha oscillations might become the dominant frequency of stimulus processing: It was shown that in monkey primary auditory cortex, periods of strong phase entrainment alternate regularly with periods of high alpha power (Figure 2C). Bursts of gamma activity and multi-unit activity (an index of neuronal firing) were coupled to the dominant oscillation: To the entrained phase when phase entrainment was strong, and to the alpha phase when alpha power was high, but entrainment was weak. Detection of deviants in an auditory sequence was significantly better in the state of strong phase entrainment than in an assumed “alpha-mode,” indicating that the auditory system might be “decoupled” from external input whenever alpha power is high. Indeed, in contrast to the visual system, where target detection depends on the alpha phase (Busch et al., 2009), auditory detection is independent of the oscillatory phase in quiet (Zoefel and Heil, 2013; Figure 2A), but this dependence can be introduced when the auditory background or electrical stimulation is rhythmic (Henry and Obleser, 2012; Neuling et al., 2012; Ng et al., 2012; Henry et al., 2014). Evidence for these two auditory modes can also be found in the data presented by Keitel and Gross (2016): The “spectral fingerprints” reported for auditory cortex include a peak at alpha frequency during rest which is replaced by peaks at slower frequencies during active listening. Moreover, (reduced) alpha power in the auditory system has been linked with the perception of illusionary phenomena, such as the Zwicker tone, an illusionary tone that is perceived for several seconds after the offset of broadband noise with a spectral gap (Leske et al., 2014). Finally, using intracranial recordings in human auditory cortex and an experimental protocol during which expectations had to be updated continuously, Sedley et al. (2016) showed that alpha power is related to the confidence (or precision) of their listeners' predictions (and thus related to internal processes) but not necessarily to the stimulus input itself.

It is unclear how the dominant frequency of stimulus processing changes if no regular structure is present in the input but attention is focused on the auditory environment. We emphasize that the switch between “entrainment-mode” and “alpha-mode,” as described above (Lakatos et al., 2016), has so far only been demonstrated during rhythmic stimulation. It was speculated that the “alpha-mode” can be activated—despite the regular stimulation—due to lapses in attention to external stimuli, leading to an increase of internal attention (an idea that was formulated already by Ray and Cole, 1985), in agreement with the other studies cited above (Leske et al., 2014; Pachitariu et al., 2015; Keitel and Gross, 2016; Sedley et al., 2016). However, in principle, a dominance of the alpha band when external input is (supposedly) ignored—and therefore virtually “*absent*” for the brain—might also mean that the alpha band dominates in the “true” *absence* of regular input. Indeed, a recent study demonstrated a relationship between MEG alpha power and the detection of non-rhythmic (i.e., unpredictable) auditory near-threshold stimuli (Leske et al., 2015). Furthermore, EEG alpha power seems to be altered when presented speech is made less rhythmic (i.e., less predictable; Kayser et al., 2015). Thus, one possibility is that in the auditory system, the switch from “entrainment-mode” to “alpha-mode” can be generalized to a larger scheme and corresponds to a switch in processing mode for regular vs. irregular stimulation. An alternative that needs to be tested is that the auditory system changes to a continuous processing mode in which sampling mechanisms of neural oscillations are suppressed. This notion was described in detail in the opinion paper by Schroeder and Lakatos (2009) and based on studies reporting a suppression of low-frequency power (and enhanced gamma-activity) in experimental paradigms where continuous vigilance is required (e.g., Fries et al., 2001). Nevertheless, these studies reported data from a specific part of the visual hierarchy (V4) and it remained unclear how the auditory system operates when no rhythmic input is present. We therefore acknowledge that the notion “entrainment vs. alpha vs. continuous mode” is speculative and discuss it in more detail in Box 2.

Box 2 **CTD: Speculations, open questions and how to test them.**

**Entrainment vs. Alpha in the Auditory System**

- The “alpha mode” might reflect a more general mode of processing that is always activated when no rhythm can be detected in the auditory environment. An alternative would be a suppression of most oscillatory sampling mechanisms when auditory attention is focused on a non-rhythmic stimulation. Both speculations must be underlined with experimental evidence. For instance, similar analyses as in Lakatos et al. (2016) might be applied in an experimental paradigm in which no regular structure is present at the input level. Intracranial recordings might be appropriate in this case, as activity in auditory cortices is, due to their nestled structure in the lateral sulcus, difficult to measure using superficial methods, such as EEG. An increase in alpha or entrained activity for irregular vs. regular stimulation, respectively, might be taken as evidence for the “alpha vs. entrainment” hypothesis described here. Another interesting approach would be the replication of previous experiments on the dependence of auditory stimulus detection in quiet on the phase of neural oscillations that so far resulted in negative results (Zoefel and Heil, 2013; VanRullen et al., 2014; Figure 2A), combined with an independent visual task on which the attention of the subjects is focused. The latter experimental manipulation would result in an absence of attention for the auditory stimulation. According to the hypothesis presented here, this lack of attention might provoke an increase of alpha activity in the auditory system. It remains to be tested whether this would result in a dependence of auditory detection on the phase of the alpha band, or if the “alpha mode” (as explained above) goes in line with a de-coupling from external events in order to avoid interference with the assumed internal processing. In the latter case, we would see an independence of auditory target detection from oscillatory activity as described previously (Zoefel and Heil, 2013).
- As mentioned above, the brain seems to be able to switch into its “alpha mode” even though rhythmic stimulation is present. It has been speculated that this switch might reflect a change from external to internal attention (Lakatos et al., 2016), but evidence for this suggestion is lacking. It needs to be determined why this is the case, and what might be a trigger for this switch. Furthermore, it needs to be clarified whether the two “modes” operate on different hierarchical levels of processing.
- It is possible that the mode of operation depends on the level of vigilance, with a system operating in a “continuous mode” during high vigilance, suppressing all oscillatory processes and taking up stimuli continuously (i.e., non-rhythmically, to avoid loss of information), and in an “alpha mode” during low vigilance where loss of information is not critical. Experimental paradigms requiring sustained attention (i.e., high vigilance) could be compared with less-demanding ones to test this idea.
- Another possibility would be a simultaneous operation of the two “modes”, but at different hierarchical levels: An “alpha mode” in higher-level auditory regions (potentially decoupled from sensory processes, reflecting internal attention) and a “continuous mode” in early auditory regions (e.g., A1). In this case, it needs to be determined how the two co-existing modes can communicate, for instance when a salient stimulus reaches a certain threshold and triggers a switch back to a mode of external attention or high vigilance.

Is this duality of oscillatory stimulus processing “modes” unique to the auditory system? Here, we argue that this is not the case, a notion that leads us back to the differentiation between “occipital alpha” and “frontal alpha” for the visual system (introduced by VanRullen, 2016). It has been argued before that the “classical” (occipital 10-Hz) alpha might serve the purpose of “saliency detection” (Jensen et al., 2012): The higher the alpha amplitude, the lower overall neuronal excitability, and the more difficult for a stimulus to reach consciousness. Thus, in an *unattended* visual scene (which leads to an increased alpha amplitude, as outlined above), occipital alpha might at the same time enable functional deactivation, but, given that an unattended stimulus is salient enough, also enable the system to switch attention to a potentially important event. This “occipital alpha” mode might be similar to the auditory “alpha mode” described in the previous paragraph: In the absence of attention, both systems might switch to a mode that is relatively independent from stimulation, and this switch can be reversed by an event that is salient enough to overcome the inhibitory effect of an increased alpha amplitude. In contrast, attention seems to be a prerequisite for a modulation of performance by “frontal alpha” in vision: Visual detection only depends on the EEG phase at 7 Hz (Figure 1B) if the stimulus is attended (Busch and VanRullen, 2010), and the observed periodicity in reaction time after a cue (Figure 1D) depends on the attended visual hemifield (Landau and Fries, 2012). Similarly, VanRullen et al. (2007) demonstrated that human psychometric data from an attentionally demanding task can be described best by a model in which attention samples input sequentially at a frequency of ~7 Hz. Therefore, only stimuli that are located in the focus of visual attention seem to be sampled at a frequency of 7–8 Hz, and this sampling frequency is independent of stimulus input (see Box 1 for further discussion). As developed above, this is in clear contrast to the auditory system where, in the presence of attention, the adaption (i.e., phase entrainment) to the frequency of stimulation seems to be a prerequisite for efficient stimulus processing.

## Further speculation, summary and conclusion

Table 1 summarizes the proposed contrasts between the visual and the auditory system in terms of oscillatory mechanisms involved in stimulus processing and selection. Some properties might be common across all systems: Neural oscillations can be used as a tool for attentional selection, and both oscillatory power and phase can be used to gate stimulus input. Changes in power might reflect a tonic suppression of processing (e.g., in a region that is currently not involved in stimulus processing) and/or change the effectiveness of the phase of an oscillation, cycling between moments of amplification and suppression. In the absence of attention, an “alpha-mode” (“occipital alpha” in the visual system) seems to be present in both systems, and is associated with a state that is decoupled from external stimulation and in which only very salient events can overcome the increased alpha amplitude and reach consciousness. Indeed, a recent study by Haegens et al. (2015) demonstrated alpha band activity in primary cortical regions of the macaque across all modalities.

**Table 1:** **Summary of mechanisms of stimulus selection and processing in the visual and auditory systems, including the hypotheses made in this article**.

	**Visual system**	**Auditory system**
Dominant frequency of processing	Alpha band (7–13 Hz): Differentiation into occipital alpha (~10 Hz) and frontal alpha (~7–8 Hz) is likely If stimulation is rhythmic and attended: Frequency of stimulation, but bias for occipital alpha If attention is absent or directed internally: Occipital alpha If stimulation is non-rhythmic and attended: Frontal alpha	Changes with respect to stimulation If stimulation is rhythmic and attended: Frequency of stimulation, but bias for slower frequencies (~1–8 Hz), as they are most prominent in natural stimuli If attention is absent or directed internally: Alpha band If stimulation is non-rhythmic and attended: Alpha band or non-oscillatory (“continuous”) processing
Adjustment to environment	Yes, but might be adjustment rather than entrainment	Yes, alignment of oscillatory phase with the rhythmic stimulus (phase entrainment) Unclear whether oscillations adjust if stimuli are predictable but non-rhythmic

However, there are differences between the visual and auditory systems: The oscillatory entrainment to rhythmic stimulation seems to be a fundamental feature of the auditory system, probably evolved due the rhythmic nature of the auditory environment. Indeed, the tendency to synchronize with auditory rhythms is ubiquitous: We sing, we dance, we clap in response to music or even to a simple beat (Nozaradan, 2014). Importantly, this phenomenon is much less pronounced for the visual system: For instance, the urge to dance is significantly lowered when watching someone dancing without the corresponding sound. Thus, although in principle the visual system also seems to be able to entrain, the adjustment of power and phase might be more important in this system—visual stimuli are often predictable, but rarely rhythmic. Interestingly, and in line with this notion, it has been shown that the auditory system is superior to the visual one when movement has to be synchronized with a rhythmic sequence in either or both modalities (Repp and Penel, 2002; Patel et al., 2005; Merchant et al., 2015) and auditory rhythmicity can influence the perceived flicker rate of a visual stimulus but not vice versa (Shipley, 1964; Herrmann et al., 2015). Task-irrelevant information in the auditory system impairs visual processing more strongly than vice versa if this information is of temporal nature (Guttman et al., 2005). Thus, although visual stimuli can in principle influence auditory processing and perception (potentially using alpha oscillations; Thorne et al., 2011; van Wassenhove and Grzeczkowski, 2015) and do so even more prominently if rhythm is involved (Ten Oever et al., 2014), a multitude of findings indicates that the auditory system dominates the visual one in the time domain (an extensive summary of the literature on this conclusion is provided in Grahn, 2012). Finally, a simple cue (*without* rhythmic component involved) is sufficient to introduce the mentioned periodic fluctuations in visual performance (Landau and Fries, 2012; Song et al., 2014; Zoefel and Sokoliuk, 2014), and recent findings suggest that the latter are present even in the absence of stimulation (Busch et al., 2009; Busch and VanRullen, 2010; Landau et al., 2015). The situation seems to be different for the auditory system, where similar periodic fluctuations in performance have been reported only after the offset of a *rhythmic* stimulus (Hickok et al., 2015) and possibly cannot be observed in the absence of rhythmicity (e.g., after a single cue; Zoefel and Heil, 2013; VanRullen et al., 2014).

In the presence of attention, stimulus processing in the visual system might be focused on the (“frontal”) alpha band (see Box 1), irrespective of the frequency of stimulation, whereas the auditory system adapts its dominant frequency of processing to that of the environment. Speculatively, if stimulation is non-rhythmic, the auditory system might operate in the alpha rhythm as well (see Box 2). However, whereas the visual alpha rhythm(s) might subsample sensory regions even when stimulus timing is unpredictable, it is possible that the auditory alpha is decoupled from sensory processes; in this way, the auditory system can avoid a loss of information that occurs when subsampling is applied to rapidly fluctuating auditory information with unknown timing. An alternative during unpredictable event timing in the auditory system would be a “continuous mode” of stimulus processing in which most oscillatory sampling mechanisms are suppressed. We note that “alpha mode” and “continuous mode” might even co-exist, but at different stages of auditory processes, or depend on the level of vigilance (see Box 2).

Neural oscillations are a powerful tool of the brain to prioritize and select relevant information while ignoring distracting input. This article summarizes the current state-of-the-art and provides several proposals that can be systematically tested and extended. Future studies and theories are indispensable to advance this exciting field of research.

## Author contributions

All authors listed, have made substantial, direct and intellectual contribution to the work, and approved it for publication.

### Conflict of interest statement

The authors declare that the research was conducted in the absence of any commercial or financial relationships that could be construed as a potential conflict of interest.

## Footnotes

^1^Note that the term “stimulus processing” is relatively general and can include a multitude of processes. In this paper, we focus on top-down or “high-level” mechanisms involved in stimulus processing, such as attention, selection, or prediction. Here, we include “environmental subsampling” (described in text) as a top-down process, as it is a mechanism initiated by the brain.

^2^We here note that the term “frontal” only refers to the observed EEG topography (e.g., Figure 1B), without any claims about the location of the underlying generators. Although some studies demonstrated an important role of frontal regions, such as the Frontal Eye Field (FEF), for alpha oscillations (Marshall et al., 2015; Popov et al., 2017), it remains unclear how these findings are related to the “frontal alpha” topography typically observed. Further studies are necessary to answer this question.

^3^We note here that, for the visual system, saccades introduce “chunks” of input arriving at a frequency of ~2-3 Hz (Otero-Millan et al., 2008) that could be considered “snapshots” of the environment and result in a temporal structuring of the visual input as well. However, we emphasize that saccades are *initiated* by the brain: The timing of incoming information is thus known in advance—e.g., via feedback from the motor system. Therefore, we argue that, in the visual system, it might not be necessary to adapt stimulus processing to the input *per se*, but rather to the (rather irregular) scanning of the environment introduced by eye movements: Indeed, there is evidence that the oscillatory phase and eye movements are linked (Hogendoorn, 2016; McLelland et al., 2016). Moreover, as the visual input “changes” every ~300–500 ms (induced by a saccade) but is rather stable within this time interval, it is not essential to process (or sample) the input at the moment of the saccade (it can be processed anytime within the ~300–500 ms interval). At the same time, this might be a reason why the visual “sampling rhythm” (assumed here as ~10 Hz), is faster than the saccadic rate: In this case, even “blind” sampling would not result in a loss of information (i.e., in the loss of one of the 300–500-ms “chunks”). Finally, we note that discrete sampling (via neural oscillations) in the visual system might even have evolved as a “shortcut” to generate “snapshots” of the environment without the metabolic costs of eye movements (for similar ideas, see Fries, 2015; VanRullen, 2016).
